# Polymer Morphological Change Induced by Terahertz Irradiation

**DOI:** 10.1038/srep27180

**Published:** 2016-06-07

**Authors:** Hiromichi Hoshina, Hal Suzuki, Chiko Otani, Masaya Nagai, Keigo Kawase, Akinori Irizawa, Goro Isoyama

**Affiliations:** 1 Terahertz Sensing and Imaging Research Team, RIKEN, 519-1399 Aramaki-Aoba, Aoba-ku, Sendai, Miyagi, 980-0845, Japan; 2 Graduate School of Engineer Science, Osaka University, 1-3 Machikaneyama, Toyonaka 560-8531, Japan; 3 Institute of Scientific and Industrial Research (ISIR), Osaka University, 8-1 Mihogaoka, Ibaraki, Osaka, 567-0047, Japan

## Abstract

As terahertz (THz) frequencies correspond to those of the intermolecular vibrational modes in a polymer, intense THz wave irradiation affects the macromolecular polymorph, which determines the polymer properties and functions. THz photon energy is quite low compared to the covalent bond energy; therefore, conformational changes can be induced “softly,” without damaging the chemical structures. Here, we irradiate a poly(3-hydroxybutylate) (PHB) / chloroform solution during solvent casting crystallization using a THz wave generated by a free electron laser (FEL). Morphological observation shows the formation of micrometer-sized crystals in response to the THz wave irradiation. Further, a 10−20% increase in crystallinity is observed through analysis of the infrared (IR) absorption spectra. The peak power density of the irradiating THz wave is 40 MW/cm^2^, which is significantly lower than the typical laser intensities used for material manipulation. We demonstrate for the first time that the THz wave effectively induces the intermolecular rearrangement of polymer macromolecules.

As the higher-order conformation determines the physical, chemical, and biological properties of polymers, technology to control macromolecular polymorphs is an important research topic for material science. It has been shown that high-power laser irradiation can perturb polymer morphology. For example, polymer nucleation^1^ and micelle aggregation^4^ via Nd:YAG laser irradiation focused by an objective lens has been demonstrated. Further, light-induced protein crystal formation has been achieved using tightly focused femtosecond lasers to irradiate a protein solution (10^10^−10^11^ W/cm^2^)^5^,^6^ Thus, laser irradiation has the potential to become a new tool for novel macromolecular polymorphism creation.

Recent remarkable progress in the development of high-power terahertz (THz) sources is introducing the possibility of material structure manipulation^7^,^8^ Pump-probe experiments using THz pulsed lasers have facilitated observation of various picosecond transient phenomena, such as the ionization of electrons induced by the ponderomotive force^9^,^10^, the THz-induced insulator–metal phase transition^11^, molecular alignment in the gas phase^12^,^13^, and highly excited intermolecular vibration via ladder climbing^14^. An intense THz wave can influence the molecular and carrier dynamics of a material; however, it is difficult to produce a fixed material structure in this manner, because of the rapid relaxation of the excited state to thermal equilibrium. An irreversible process is required to maintain the instantaneous structure following THz irradiation.

In this study, we irradiate a poly(3-hydroxybutylate) (PHB) / chloroform solution with THz waves during solvent casting crystallization. PHB is one of the polymers for which the vibrational modes in both the infrared^15^ and THz regions^16^,^17^ are well assigned. In the infrared spectra (C = O str. region), the crystalline and amorphous structures are clearly distinguishable via the frequency shift, which enables quantitative analysis of the crystallinity. In the THz region, some vibrational modes are coupled with the intermolecular hydrogen bonds. As the THz-wave photon energy corresponds to the vibrational energy of the polymer hydrogen bonds^16^,^17^,^18^, the THz waves can excite intermolecular motion effectively. Moreover, the THz photon energy is quite low compared to that of the covalent bonds; thus, the conformational change occurs “softly,” without damaging the chemical structure. The obtained polymer structure can be fixed following the irradiation, because of the irreversibility of the solvent casting crystallization.

The experimental setup used in this study is shown in Fig. 1, and further details of the experimental procedure are available in the Methods section. Figure 2 shows optical microscope images, IR spectra, and IR images of PHB films grown with and without THz irradiation (center wavelength λ  = 57 μm, average macropulse energy *E*_THz_ = 3.9 mJ). Figure 2(a) shows the images obtained by a low-magnification polarized microscope in the reflection setup, with the polarizers set to a cross-Nicol configuration. No significant differences are apparent with sample rotation, for both the specimens with and without THz irradiation; this indicates the absence of birefringence. The image contrasts are primarily due to scattering by the micrometer-sized structures in the samples. Figure 2(b) shows magnified images of the sample centers obtained using a laser confocal microscope (Olympus: OLS3100) in the transmission setup. A clear morphological difference can be observed between the two samples. Without THz irradiation, a few micrometer-sized structures are uniformly distributed throughout the observed area, in which a number of dark, micrometer-sized, curved structures are randomly tangled (I and II in Fig. 2(b)). According to the results of previous morphological studies^19^, the random and complex structures can be understood as multi-shape early-grown spherulite structures. On the other hand, the THz-irradiated sample is composed of bright, needle-shaped structures with ~1-μm thickness (III) and dark structure aggregates (IV). The sizes and shapes of the needle-shaped structures are quite similar to those of PHB single crystals slowly grown from a dilute solution^20^. The round-shaped aggregates in Fig. 2(b) (IV) appear to be spherulite structures comprised of smaller crystals. The existence of these two structures strongly suggests that this specimen has higher crystallinity than the un-irradiated material.

To estimate the crystallinity of the PHB films quantitatively, IR spectroscopic images were obtained using an FT-IR microscope (JASCO: FT/IRT-7200). Figure 2(c) shows the absorption spectra measured at the PHB film centers. It is known that the C = O stretching band reflects the PHB crystalline structures^15^. Specifically, crystalline PHB exhibits a sharp peak at 1722−1723 cm^−1^, while amorphous PHB has a broad absorption band at ~1740 cm^−1 ^15. The dashed lines in Fig. 2(c) show the result of a least-squares fitting of two Gaussian functions. Herein, the crystallinity, i.e., the molar ratio between the crystalline and amorphous PHB, is defined by the ratio of the integrated absorption intensities of those two peaks. Thus, the crystallinity values in Fig. 2(c) are 37% and 57% without and with THz irradiation, respectively. The PHB crystallinity induced by the solvent casting crystallization is remarkably lower than that of typical melt-crystallized samples (~55%)^21^, suggesting that the PHB metastable structure changes to a stable polymorph under THz wave irradiation.

Figure 2(d) shows the spatial distributions of the crystallinity for both the un-irradiated and irradiated specimens, as determined via 2D imaging spectra measured over an area of 7.4 × 7.4 mm^2^. To visualize the crystalline distribution, the intensity ratio of the absorbance between 1722 and 1743 cm^−1^ was plotted. For the specimen subjected to THz beam irradiation, the center area of ~5-mm diameter was clearly transformed to a high-crystallinity region. Note that the size and shape of that area conform to the THz beam pattern at the sample position.

From the results shown in Fig. 2, we can conclude that the PHB crystallinity was improved by the THz beam irradiation. As no significant changes were observed in the other regions of the IR spectra, the PHB chemical structure was not damaged by the THz irradiation and only the higher-order conformation was changed. Note that similar phenomena, known as laser-induced crystallization, have been reported in various studies since the 1990 s^22^. In those cases, the nucleation from the solution was accelerated under the tightly focused laser pulses, in which the energy fluence was of the order of GW/cm^2^. Under such conditions, it was possible for the nucleation to be triggered by various non-linear phenomena, such as chemical reactions with multi-photon excitation, clustering with photon pressure, and molecular ionization by ponderomotive forces. On the other hand, the peak power of the THz micropulses in this study is ~40 MW/cm^2^ and, therefore, non-linear effects are almost negligible.

The simplest explanation of the observed crystallization involves the thermal effect, because polymer crystallization is easily affected by slight changes in the sample temperature. We measured the average sample temperature during the THz wave irradiation process by dipping a K-type thermocouple directly into the sample. At the center of the THz beam spot, the temperature increase was less than 1 °C. In addition, we crystallized PHB films without THz irradiation by changing the temperature from 22 to 32 °C, and the corresponding FT-IR spectra indicated that the difference in the crystallinity was negligible in this temperature region. Thus, the increased crystallinity observed in the THz-irradiated specimens in this study was not caused by the average temperature increase induced by the THz irradiation.

Another explanation is that the THz wave directly excites intermolecular vibration and induces a conformational change. In Fig. 3, the sample crystallinity is plotted as a function of the THz energy density and for different THz wavelengths. A clear correlation can be seen between the THz energy density and crystallinity; however, the dependence on the THz wavelength is unclear. Figure 4 shows the THz absorption spectra of (a) crystalline PHB^23^ (b) a PHB solution with chloroform, and pure chloroform. The results for the 57- and 100-μm THz wavelengths overlap the vibrational band of the crystalline PHB, with the transition dipole moments being parallel to the *b*- and *c*- crystal axes, respectively^17^ Note that, if the crystallization were induced by excitation of those vibrational modes, the on- and off-resonance of the wavelength would result in significant differences in the sizes and orientations of the different wavelengths shown in Fig. 3. However, this is not the case.

Based on the above results, the majority of the THz energy radiated onto the sample is absorbed by the dissolved PHB and chloroform. The absorbance of the PHB solution with chloroform and that of pure chloroform are shown in Fig. 4(b). The THz wave energy is primarily absorbed by the chloroform, and the instantaneous increase in the temperature due to the micropulse may cause shockwave generation. The resultant chain of shockwaves may trigger a structural change. Thus, the conformational change in the PHB from the metastable (lower crystallinity) to the stable (higher crystallinity) structure may be triggered by this type of weak perturbation. In fact, it has recently been reported that the crystallization of acetaminophen from the metastable phase is induced by ultrasonic irradiation^24^ Similar phenomena may occur in PHB solutions. Typically, laser-induced shockwave generation is observed for laser energy fluence of the order of GW/cm^2^ in the near infrared region but, in our case, the THz beam energy fluence is more than 100 times lower. When the near infrared lasers are employed, the photon energy is too far from the energy of the intermolecular motion. On the other hand, the THz frequency overlaps with the intermolecular vibrational modes of solvent. The THz wave energy can be effectively transferred to shockwave generation, because the THz wave irradiation can excite the intermolecular motion directly.

Although the mechanism behind the THz-radiation-induced morphological change remains unclear, we can change the intermolecular conformation of bulk materials “softly,” without damaging the chemical structures. This advantage will facilitate material control of “fragile” molecules such as bio-macromolecules. We believe that the use of THz irradiation may constitute a new method that is applicable not only to polymer science, but also to biological science, as a means of discovering new functional materials.

## Methods

**Figure 1:**
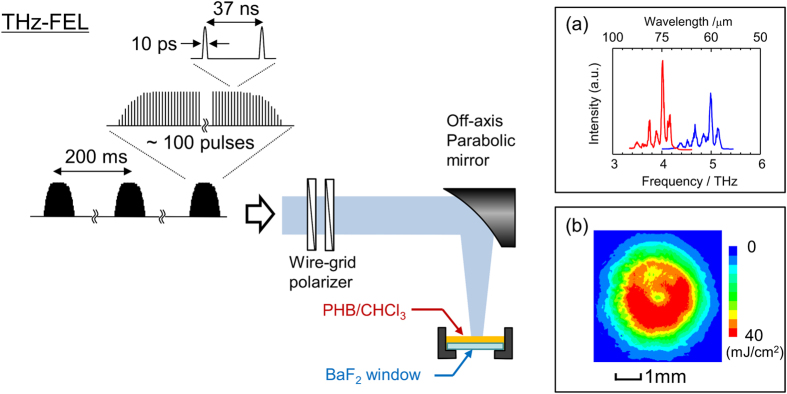
Schematic of experimental setup. The THz-free electron laser (FEL) output 5-Hz macropulses composed of 100 micropulses with 10-ps duration. The THz-FEL output was attenuated by a pair of wire-grid polarizers and focused by an off-axis parabolic mirror (effective focal length (EFL) = 102 mm). The sample was dropped on the BaF_2_ window, which was placed at a 25-mm offset from the focal point. Insets: (**a**) Typical spectral profile measured at λ = 60 and 75 μm; (**b**) Typical beam pattern at sample position, measured at λ  = 70 μm.

A THz-free electron laser (FEL) at the Institute of Scientific and Industrial Research (ISIR), Osaka University, was used as the THz beam source. Details on the THz-FEL can be found elsewhere^25^,^26^ The THz-FEL output macro-pulses with a 5-Hz repetition rate, composed of ~100 micropulses with 10-ps duration. The output THz pulse contained several wavelength components within an ~1 THz bandwidth (Fig. 1(a)), and the center wavelength λ was tuned by changing the wiggler gap distance. Fig. 1 is a schematic of the experimental setup. The THz beam from the FEL was focused by an off-axis parabolic mirror with 102-mm focal length and the sample was offset from the focal point by 25 mm, so that the THz radiation was loosely focused on the specimen. The average power of the macropulse *E*_THz_ was measured by a pyroelectric detector (Coherent Inc.: J-25MB-LE) and this value was adjusted by a pair of wire-grid polarizers placed in the optical path. The typical beam pattern (λ = 70 μm; *E*_THz_ = 4.5 mJ) at the sample position was monitored by a THz camera (NEC: IRV-T0831; Fig. 1, inset). The beam radius was approximately 4 mm full width at half maximum (FWHM) and the macropulse energy density was estimated to be ~40 mJ/cm^2^ at the beam center; this corresponds to an ~40-MW/cm^2^ maximum peak power for the micropulse.

The THz beam was employed during the formation of the poly(3-hydroxybutylate) (PHB) film sample from a dilute solution. 200 μl of PHB (Aldrich Corp.) solution with chloroform (3.9 mg/ml) was dropped on a BaF_2_ window, which had 19-mm aperture size and was placed horizontally in an Al cell. The cell was at room temperature (24 °C) and a PHB film sample was grown on the window through 30-min evaporation of the chloroform solution. During the evaporation, the center of the sample cell was continuously irradiated by the THz beam. Following formation of the PHB film, the BaF_2_ window was detached from the cell and observed under polarized, laser confocal, and Fourier transform infrared (FT-IR) spectroscopy microscopes. PHB film samples were also prepared without THz irradiation, using the same growth process.

## Additional Information

**How to cite this article**: Hoshina, H. *et al.* Polymer Morphological Change Induced by Terahertz Irradiation. *Sci. Rep.* **6**, 27180; doi: 10.1038/srep27180 (2016).

## Figures and Tables

**Figure 2:**
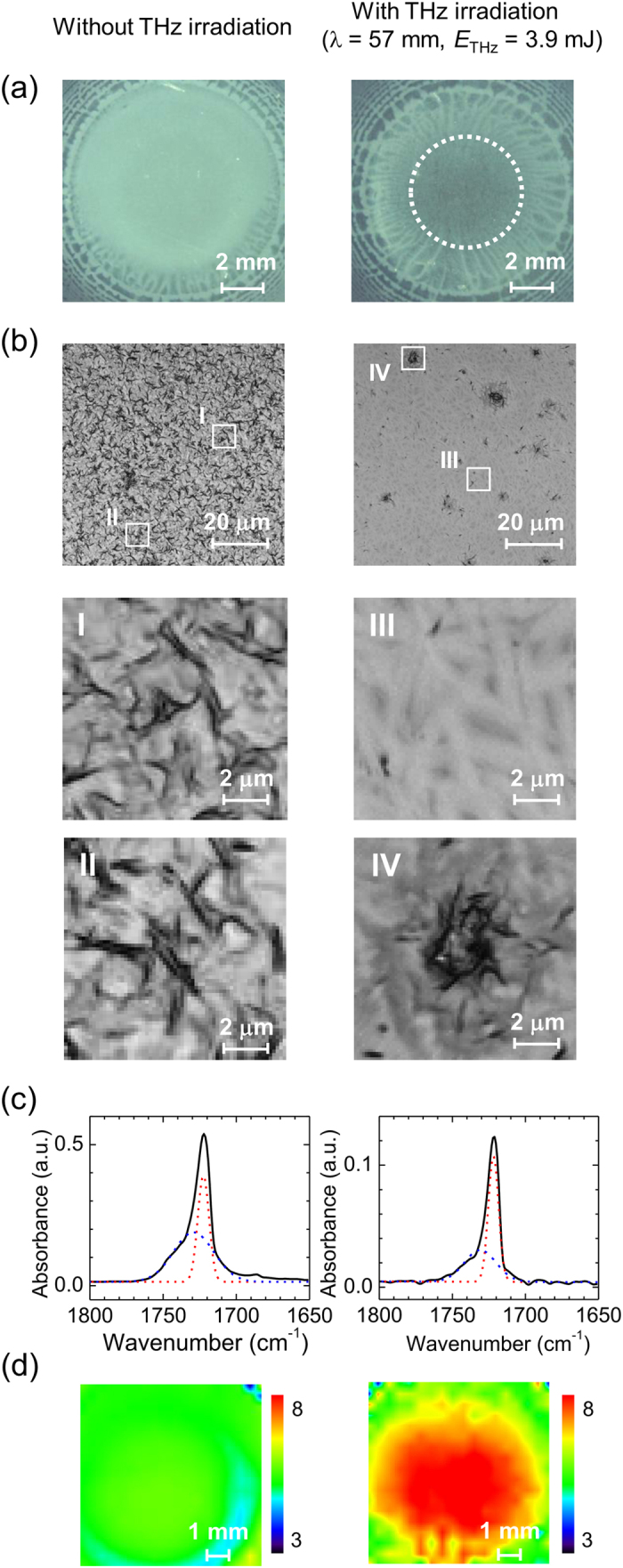
Optical images, IR spectra, and IR images of PHB films grown without and with THz irradiation (λ = 57 μm, *E*_THz_ = 3.9 mJ). (**a**) Low-magnification reflection images obtained by polarized microscope with cross-Nicol setup. The dashed line shows the irradiated THz wave beam size. (**b**) Transmission microscope images obtained using confocal laser microscope. Images (I–IV) are 10×-magnified. (**c**) IR absorption spectra measured at sample centers. The dashed lines show the result of a least-squares fitting with Gaussian functions. (**d**) 2D imaging plots of absorbance intensity ratios between 1722 and 1743 cm^−1^.

**Figure 3:**
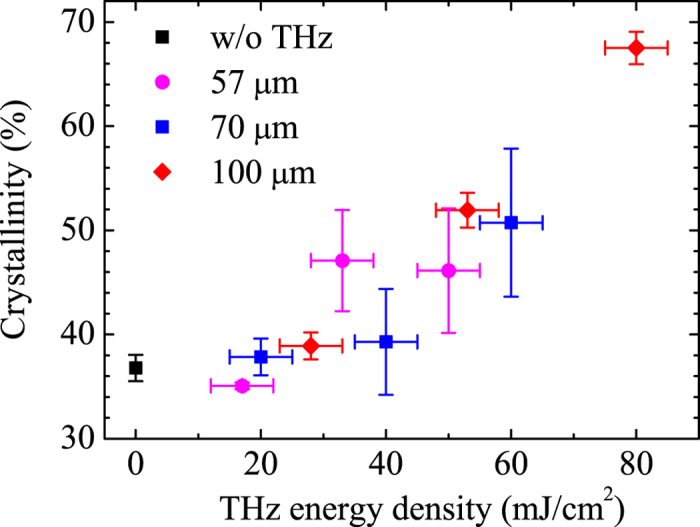
PHB film crystallinity with THz irradiation, as a function of macropulse THz energy density and for different wavelengths.

**Figure 4:**
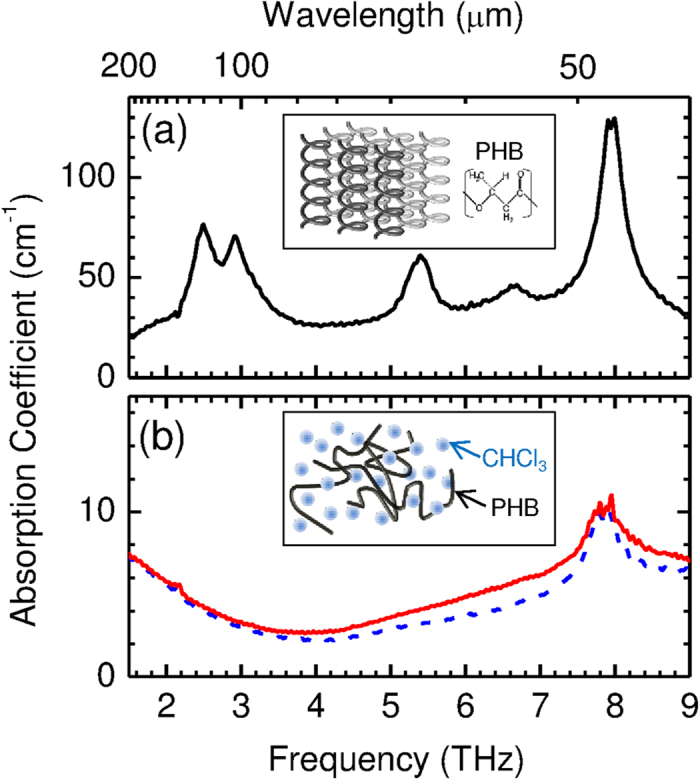
THz absorption spectra of (**a**) crystalline PHB, (**b**) PHB solution with chloroform (solid), and pure chloroform (dashed). The insets are schematics of the intermolecular structure.
